# The Decline in the Birth-Rate

**Published:** 1913-08-30

**Authors:** 


The Hospital

The Workers' Newspaper of Administrative Medicine and Institutional Life, Administration, National Insurance and Health.

No. 1418, Vol. LIV. SATURDAY, AUGUST 30, 1913. PRICE ONE PENNY

### THE DECLINE IN THE BIRTH-RATE.

Professor Karl Pearson has earned for himself the title of "Corrector of the Errors of the Profession," for he has so ably and categorically demonstrated the prevailing fallacies of medical men and medical teachers that we have come to take any remark he makes with the respect to which an eminent authority is entitled. No one can for a moment doubt that as an explainer of statistics Professor Pearson is unrivalled. No one, again, can reasonably maintain that the profession knows how to deal with statistics. Conclusions drawn in the past from an erroneous use of available medical statistics have resulted in all sorts of mistakes. The most common of these is probably the consideration of the birth-rate by itself without relating and comparing its figures, for instance, with those of the death-rate, without which no sane conclusions can be arrived at, as Havelock Ellis has so often and pertinently shown.

Against this tendency Professor Pearson has worked vigorously, and the profession is indebted to him for his usefulness as a corrective factor in—to mention only one instance—the absurd over-representation of the case against alcohol. Now he is once more in the field to put right some errors with regard to the birth-rate. His lecture at the London School of Economics, delivered of late, is eminently one that should be carefully studied by the medical man, for it shows the lines on which reform work in the future must be carried out. Professor Pearson believes—and we incline to think that his belief is right—that the value of the child population of a nation is fixed, in general, by the physical value of the mothers. He finds that artificial feeding and the many other causes which medical men are in the habit of regarding as certain factors of infant mortality have, on the whole, less danger to the community than is to be found in a defective maternal parent vitiated by disease or by hereditary enfeeblement. This is a deduction which should be pressed home. In every civilised country the birth-rate is falling; that fact alone is not sufficient to cause any uneasiness to those who rightly understand the difference between civilisation and savagery in regard to this aspect of motherhood. With the economic factors to be considered, it is easy to see that the child-bearing period of women in civilised communities is steady contracting, and that the physical enfeeblement of women exposed to the stress and strain of modern life must inevitably lead to the physical deterioration of subsequent generations unless something more than at present is done for the benefit of the coming race. That being the case, the real question at issue is not whether the birth-rate is declining. With these factors present it is inevitable, and indeed expedient, that the birth-rate should decline, for no honest parent can afford to bring into the world children whom he cannot fit with a strong constitution and endow with that physical and social means of safeguarding their position when they are grown up. On the contrary, the question concerns not the number of births, but the value of the births recorded. A declining infant mortality must ensure that a larger number of children will reach the age of puberty; the prophylactic, ultra-humanitarian measures of to-day make it possible for the weakest and unfittest to survive along with the strongest and best. Indeed, the tendency is to safeguard the interests of the former at the expense of those of the latter, a tendency which is bound ultimately to affect the race detrimentally.

The whole matter of the birth-rate in reference to the value of the births and the status of the community cannot be settled on statistical grounds alone. Nevertheless, we are duly grateful to Professor Pearson for drawing attention to certain outstanding facts which medical men are apt to overlook. Such semi-popular lectures should make the nation alive to the facts that ought never to be forgotten, but which are, unfortunately, remembered by comparatively few public men. One of these facts is the desirability of training the rising generation so as to perfect its physique and enhance its means of resistance to factors that act adversely upon less resistant organisms. This fact should be acknowledged by every public man, and it is a great pity that the unfortunate bogey of alleged "militarism" still interferes with the due appreciation of the benefits to be derived from such physical and early training as military discipline entails. The recent pronouncement that no such training will be allowed in the public elementary schools is a deplorable testimony to the apathy and shortsightedness of a nation that still regards party politics as of far more importance than a healthy birth-rate. When Professor Pearson again lectures on the subject we commend to him to pay some attention to the position of the father. An investigation into the antecedents and environmental conditions of the father should prove interesting, and we venture to think that some of the results to be obtained from such a study ought to be of decided value to the community.

